# Supplementary material for: Joint observation in NICU (JOIN): A randomized controlled trial testing an early, one-session intervention during preterm care to improve perceived maternal self-efficacy and other mental health outcomes
Source: PLoS One. 2024 Apr 25;19(4):e0301594. doi: 10.1371/journal.pone.0301594 (PMC11045081; doi:10.1371/journal.pone.0301594)

**Supporting information captions**

**S1 Fig. Perceived Maternal Parenting Self-Efficacy – Within-group scores distributions**

Within-group scores distributions are separately displayed for the intervention group and for the control group, with statistically significant time effects in both groups.

PMP-SE: Perceived Maternal Parenting Self-Efficacy; 1-Pre: Pre-intervention’s time point; 2-Post: Post-intervention’s time point; 6-Mths: 6 months’ time points


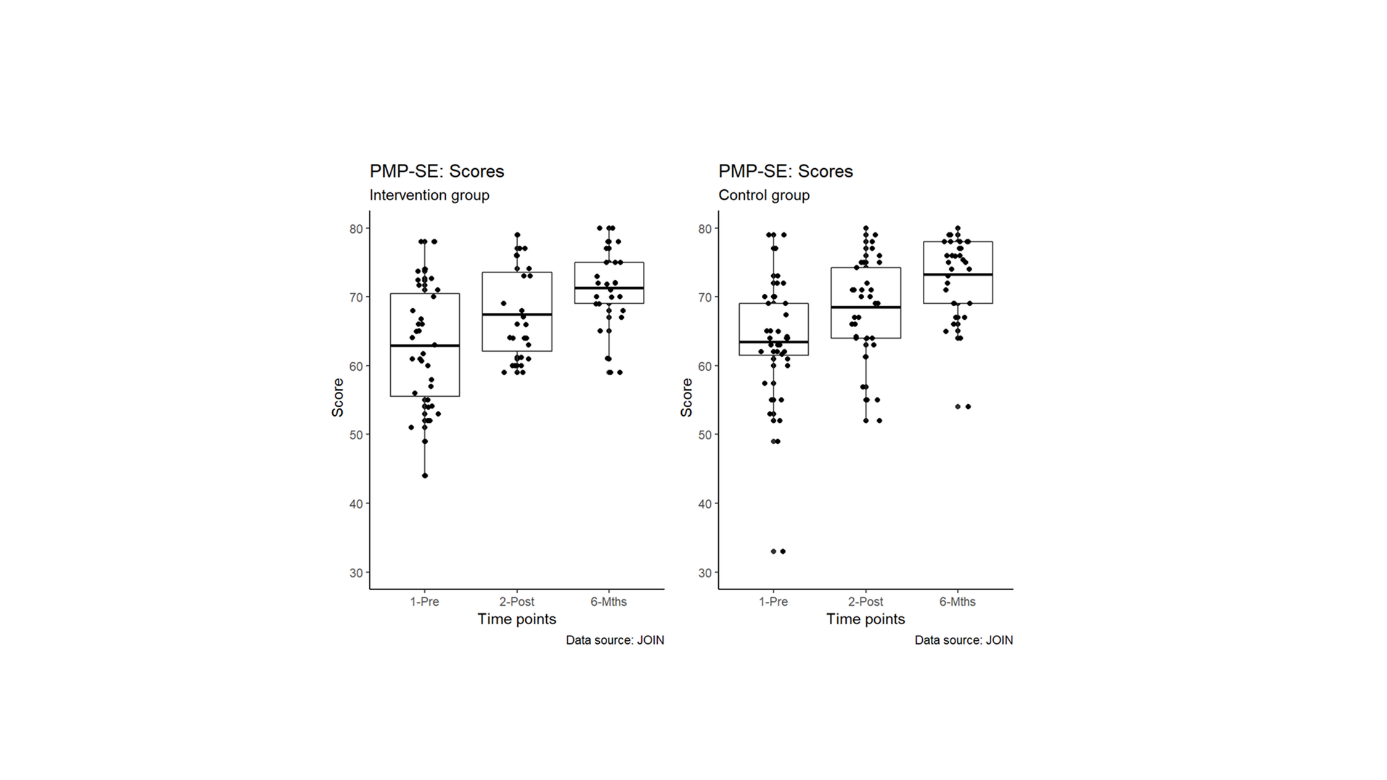

Supplement: S1 Fig — Within-group scores distributions are separately displayed for the intervention group and for the control group, with statistically significant time effects in both groups. PMP-SE: Perceived Maternal Parenting Self-Efficacy; 1-Pre: Pre-intervention’s time point; 2-Post: Post-intervention’s time point; 6-Mths: 6 months’ time points. (DOCX) [file pone.0301594.s001.docx]
