# Supplementary material for: Joint observation in NICU (JOIN): A randomized controlled trial testing an early, one-session intervention during preterm care to improve perceived maternal self-efficacy and other mental health outcomes
Source: PLoS One. 2024 Apr 25;19(4):e0301594. doi: 10.1371/journal.pone.0301594 (PMC11045081; doi:10.1371/journal.pone.0301594)
Supplement: S1 Table — (DOCX) [file pone.0301594.s002.docx]

**S1 Table. Missing values in questionnaires**

| Questionnaire | Time point | Total missing values | Max missing value in single item | Max missing value in single obs. |
| --- | --- | --- | --- | --- |
| PMP-SE | T1 | 27 (1.9%) | 5 | 5 |
|  | T2 | 14 (1.11%) | 2 | 5 |
|  | T3 | 7 (0.57%) | 2 | 3 |
| IBQ-R Total | T1 | 21 (0.8%) | 2 | 8 |
|  | T2 | 13 (0.57%) | 3 | 4 |
|  | T3 | 45 (2.1%) | 8 | 7 |
| IBQ-R Surgency | T1 | 3 (0.33%) | 2 | 2 |
|  | T2 | 4 (0.5%) | 1 | 2 |
|  | T3 | 17 (2.34%) | 6 | 2 |
| IBQ-R Negative Affect | T1 | 4 (0.48%) | 2 | 2 |
|  | T2 | 5 (0.68%) | 3 | 2 |
|  | T3 | 11 (1.58%) | 2 | 2 |
| IBQ-R Effortful Control | T1 | 3 (0.35%) | 1 | 2 |
|  | T2 | 1 (0.13%) | 1 | 1 |
|  | T3 | 9 (1.29%) | 4 | 2 |
| MOS-SS | T1 | 3 (0.56%) | 3 | 1 |
|  | T2 | 1 (0.2%) | 1 | 1 |
|  | T3 | 0 (0%) | 0 | 0 |
| PSI-Total | T1 | 80 (3.27%) | 19 | 7 |
|  | T2 | 22 (0.99%) | 5 | 5 |
|  | T3 | 23 (1.08%) | 6 | 5 |
| PSI-PD | T1 | 9 (1.06%) | 3 | 2 |
|  | T2 | 3 (0.4%) | 1 | 2 |
|  | T3 | 4 (0.56%) | 1 | 2 |
| PSI-PCDI | T1 | 5 (0.62%) | 2 | 2 |
|  | T2 | 3 (0.4%) | 2 | 1 |
|  | T3 | 0 (0%) | 0 | 0 |
| PSI-DC | T1 | 26 (3.74%) | 12 | 2 |
|  | T2 | 10 (1.39%) | 5 | 2 |
|  | T3 | 10 (1.46%) | 5 | 2 |
| HADS-Total | T1 | 5 (0.5%) | 2 | 3 |
|  | T2 | 2 (0.23%) | 1 | 1 |
|  | T3 | 1 (0.12%) | 1 | 1 |
| HADS-Anxiety | T1 | 1 (0.2%) | 1 | 1 |
|  | T2 | 2 (0.45%) | 1 | 1 |
|  | T3 | 1 (0.23%) | 1 | 1 |
| HADS-Depression | T1 | 2 (0.4%) | 1 | 1 |
|  | T2 | 0 (0%) | 0 | 0 |
|  | T3 | 0 (0%) | 0 | 0 |
| F-PSS-NICU-Total | T1 | 18 (0.81%) | 3 | 8 |
|  | T2 | 12 (0.62%) | 2 | 5 |
|  | T3 | 30 (1.73%) | 5 | 5 |
| PSS-Visual and Auditive | T1 | 1 (0.16%) | 1 | 1 |
|  | T2 | 2 (0.36%) | 1 | 1 |
|  | T3 | 5 (0.91%) | 2 | 1 |
| PSS-Baby Behavior | T1 | 3 (0.32%) | 1 | 2 |
|  | T2 | 3 (0.37%) | 2 | 2 |
|  | T3 | 7 (1.04%) | 3 | 2 |
| PSS-Parent Role | T1 | 5 (0.78%) | 2 | 1 |
|  | T2 | 1 (0.18%) | 1 | 1 |
|  | T3 | 2 (0.41%) | 1 | 1 |
| MIBS | T1 | 2 (0.37%) | 1 | 1 |
|  | T2 | 1 (0.2%) | 1 | 1 |
|  | T3 | 1 (0.21%) | 1 | 1 |
| EPDS | T1 | 5 (0.69%) | 2 | 2 |
|  | T2 | 2 (0.32%) | 1 | 1 |
|  | T3 | 1 (0.17%) | 1 | 1 |

Abbreviations: EPDS: Edinburgh Postnatal Depression Scale; F-PSS-NICU: Parental Stressor Scale: neonatal intensive care unit; HADS: Hospital Anxiety and Depression Scale; IBQ-R: Infant Behavior Questionnaire-Revised Very Short Form; MIBS: Mother-to-Infant Bonding Scale; m-MOS-SS: Modified Medical Outcomes Study Social Support Survey; PMP-SE: Perceived Maternal Self-efficacy; PSI: Parenting Stress Index; PSI-PD: Parenting Stress Index – Parental Distress; PSI-PCDI: Parenting Stress Index – Parent-Child Dysfunctional Interaction; PSI-DC: Parenting Stress Index - Difficult Child; PSS V&A: Parental Stressor Scale Visual & Auditive; PSS BB: Parental Stressor Scale Baby Behavior; PSS PR: Parental Stressor Scale Parent Role.
