# Supplementary material for: Joint observation in NICU (JOIN): A randomized controlled trial testing an early, one-session intervention during preterm care to improve perceived maternal self-efficacy and other mental health outcomes
Source: PLoS One. 2024 Apr 25;19(4):e0301594. doi: 10.1371/journal.pone.0301594 (PMC11045081; doi:10.1371/journal.pone.0301594)
Supplement: S2 Table — The results of the maternal questionnaires at baseline (pre-intervention) for both the intervention and the control groups are presented in the table showing no statistically significant between-group differences. (DOCX) [file pone.0301594.s003.docx]

**S2 Table. Between-group differences at baseline – Outcomes**

| Outcomes | Intervention | Control | p |
| --- | --- | --- | --- |
| PMP-SE score (N, M, SD) | 35, 62.93 (8.99) | 36, 63.49 (8.72) | .660 |
|  |  |  |  |
| mMOS-SS score (N, M, SD) | 36, 33.75 (5.82) | 33, 34.77 (5.64) | .380 |
|  |  |  |  |
| PSI (N, M, SD) |  |  |  |
| PSI Total scores | 34, 146.30 (17.00) | 34, 151.00 (15.00) | .380 |
| PSI – PD | 34, 47.33 (8.51) | 34, 48.64 (6.45) | .479 |
| PSI – PCDI | 34, 50.60 (6.22) | 34, 52.46 (5.32) | .132 |
| PSI – DC | 34, 48.31 (6.53) | 34, 50.04 (5.50) | .221 |
|  |  |  |  |
| HADS (N, M, SD) |  |  |  |
| HADS total scores | 36, 16.85 (6.95) | 36, 16.71 (7.21) | .933 |
| HADS Anxiety | 36, 9.24 (4.33) | 36, 9.25 (4.24) | .992 |
| HADS Depression | 36, 7.61 (3.74) | 36, 7.46 (3.93) | .910 |
|  |  |  |  |
| F-PSS-NICU (N, M, SD) |  |  |  |
| PSS Total | 36, 2.70 (0.72) | 36, 2.76 (0.65) | .705 |
| PSS V&A | 36, 2.68 (0.70) | 36, 2.61 (0.76) | .669 |
| PSS BB | 36, 2.59 (0.87) | 36, 2.66 (0.82) | .708 |
| PSS PR | 36, 2.88 (1.07) | 36, 3.06 (0.94) | .452 |
|  |  |  |  |
| IBQ-R (N, M, SD) |  |  |  |
| IBQ-Total Scores | 36, 1.05 (0.54) | 35, 1.22 (0.56) | .189 |
| IBQ-R SUR | 36, 0.61 (0.52) | 35, 0.60 (0.46) | .995 |
| IBQ-R NEG | 36, 0.71 (0.48) | 35, 0.89 (0.82) | .674 |
| IBQ-R EFF | 36, 1.86 (1.02) | 35, 2.22 (0.85) | .110 |
|  |  |  |  |
| MIBS (N, M, SD) | 33, 2.13 (2.32) | 35, 2.49 (2.31) | .369 |
|  |  |  |  |
| EPDS (N, M, SD) | 36, 10.98 (5.65) | 36, 9.66 (4.25) | .266 |

The results of the maternal questionnaires at baseline (pre-intervention) for both the intervention and the control groups are presented in the table showing no statistically significant between-group differences.

Abbreviations: EPDS: Edinburgh Postnatal Depression Scale; F-PSS-NICU: Parental Stressor Scale: neonatal intensive care unit; HADS: Hospital Anxiety and Depression Scale; IBQ-R: Infant Behaviour Questionnaire-Revised Very Short Form; IBQ-R SUR: Infant Behavior Questionnaire-Revised Surgency; IBQ-R NEG: Infant Behavior Questionnaire-Revised Negative Affect; IBQ-R EFF: Infant Behavior Questionnaire-Revised Effortful Control; MIBS: Mother-to-Infant Bonding Scale; m-MOS-SS: Modified Medical Outcomes Study Social Support Survey; PMP-SE: Perceived Maternal Self-efficacy; PSI: Parenting Stress Index; PSI-PD: Parenting Stress Index – Parental Distress; PSI-PCDI: Parenting Stress Index – Parent-Child Dysfunctional Interaction; PSI-DC: Parenting Stress Index - Difficult Child; PSS V&A: Parental Stressor Scale Visual & Auditive; PSS BB: Parental Stressor Scale Baby Behavior; PSS PR: Parental Stressor Scale Parent Role.
