# Supplementary material for: Joint observation in NICU (JOIN): A randomized controlled trial testing an early, one-session intervention during preterm care to improve perceived maternal self-efficacy and other mental health outcomes
Source: PLoS One. 2024 Apr 25;19(4):e0301594. doi: 10.1371/journal.pone.0301594 (PMC11045081; doi:10.1371/journal.pone.0301594)
Supplement: S3 Table — (DOCX) [file pone.0301594.s004.docx]

**S3** **Table. Cronbach alphas for study questionnaires**

| Outcomes | Time points | Alpha [95% CI] |
| --- | --- | --- |
| PMP-SE | Pre-Intervention | 0.926 [0.891, 0.938] |
|  | Post-Intervention | 0.915 [0.888, 0.919] |
|  | 6 months | 0.900 [0.850, 0.913] |
|  |  |  |
| PSI Total | Pre-Intervention | 0.912 [0.895, 0.926] |
|  | Post-Intervention | 0.951 [0.924, 0.964] |
|  | 6 months | 0.908 [0.873, 0.927] |
|  |  |  |
| PSI-PD | Pre-Intervention | 0.804 [0.755, 0.855] |
|  | Post-Intervention | 0.919 [0.849, 0.944] |
|  | 6 months | 0.880 [0.837, 0.900] |
|  |  |  |
| PSI-PCDI | Pre-Intervention | 0.872 [0.794, 0.904] |
|  | Post-Intervention | 0.891 [0.779, 0.936] |
|  | 6 months | 0.796 [0.716, 0.852] |
|  |  |  |
| PSI-DC | Pre-Intervention | 0.844 [0.751, 0.856] |
|  | Post-Intervention | 0.869 [0.712, 0.924] |
|  | 6 months | 0.800 [0.724, 0.829] |
|  |  |  |
| HADS-Total | Pre-Intervention | 0.829 [0.794, 0.865] |
|  | Post-Intervention | 0.876 [0.849, 0.897] |
|  | 6 months | 0.869 [0.824, 0.891] |
|  |  |  |
| HADS-Anxiety | Pre-Intervention | 0.825 [0.759, 0.871] |
|  | Post-Intervention | 0.819 [0.775, 0.852] |
|  | 6 months | 0.821 [0.758, 0.874] |
|  |  |  |
| PSS-Total | Pre-Intervention | 0.901 [0.881, 0.920] |
|  | Post-Intervention | 0.919 [0.900, 0.942] |
|  | 6 months | 0.946 [0.923, 0.956] |
|  |  |  |
| PSS V&A | Pre-Intervention | 0.779 [0.724, 0.820] |
|  | Post-Intervention | 0.774 [0.725, 0.861] |
|  | 6 months | 0.865 [0.816, 0.891] |
|  |  |  |
| PSS BB | Pre-Intervention | 0.841 [0.812, 0.865] |
|  | Post-Intervention | 0.864 [0.856, 0.880] |
|  | 6 months | 0.914 [0.909, 0.940] |
|  |  |  |
| PSS PR | Pre-Intervention | 0.872 [0.830, 0.892] |
|  | Post-Intervention | 0.912 [0.873, 0.921] |
|  | 6 months | 0.921 [0.879, 0.940] |
|  |  |  |
| IBQ-R Total | Pre-Intervention | 0.812 [0.802, 0.822] |
|  | Post-Intervention | 0.913 [0.892, 0.928] |
|  | 6 months | 0.826 [0.747, 0.854] |
|  |  |  |
| IBQ-R Surgency | Pre-Intervention | 0.598 [0.523, 0.723] |
|  | Post-Intervention | 0.791 [0.632, 0.837] |
|  | 6 months | 0.641 [0.527, 0.716] |
|  |  |  |
| IBQ-R Negative Affect | Pre-Intervention | 0.705 [0.617, 0.782] |
|  | Post-Intervention | 0.863 [0.797, 0.897] |
|  | 6 months | 0.836 [0.668, 0.869] |
|  |  |  |
| IBQ-R Effortful Control | Pre-Intervention | 0.690 [0.611, 0.742] |
|  | Post-Intervention | 0.785 [0.676, 0.853] |
|  | 6 months | 0.690 [0.590, 0.739] |
|  |  |  |
| MIBS | Pre-Intervention | 0.614 [0.526, 0.673] |
|  | Post-Intervention | 0.639 [0.528, 0.662] |
|  | 6 months | 0.707 [0.555, 0.777] |
|  |  |  |
| EPDS | Pre-Intervention | 0.808 [0.786, 0.847] |
|  | Post-Intervention | 0.883 [0.814, 0.907] |
|  | 6 months | 0.909 [0.888, 0.928] |

Abbreviations: EPDS: Edinburgh Postnatal Depression Scale; F-PSS-NICU: Parental Stressor Scale: neonatal intensive care unit; HADS: Hospital Anxiety and Depression Scale; IBQ-R: Infant Behavior Questionnaire-Revised Very Short Form; MIBS: Mother-to-Infant Bonding Scale; m-MOS-SS: Modified Medical Outcomes Study Social Support Survey; PMP-SE: Perceived Maternal Self-efficacy; PSI: Parenting Stress Index; PSI-PD: Parenting Stress Index – Parental Distress; PSI-PCDI: Parenting Stress Index – Parent-Child Dysfunctional Interaction; PSI-DC: Parenting Stress Index - Difficult Child; PSS V&A: Parental Stressor Scale Visual & Auditive; PSS BB: Parental Stressor Scale Baby Behavior; PSS PR: Parental Stressor Scale Parent Role.
