# Supplementary material for: Joint observation in NICU (JOIN): A randomized controlled trial testing an early, one-session intervention during preterm care to improve perceived maternal self-efficacy and other mental health outcomes
Source: PLoS One. 2024 Apr 25;19(4):e0301594. doi: 10.1371/journal.pone.0301594 (PMC11045081; doi:10.1371/journal.pone.0301594)
Supplement: Attachment — Submitted filename: renamed_52d50.docx [file pone.0301594.s005.docx]

# COMMENTS – Manuscript PONE-D-23-00084

Title: “Joint observation in NICU (JOIN): a randomized controlled trial testing an early, one-session intervention during preterm care to improve perceived maternal self-efficacy and other mental health outcomes”

**Important note: This review pertains only to ‘statistical aspects’ of the study and so ‘clinical aspects’ [like medical importance, relevance of the study, ‘clinical significance and implication(s)’ of the whole study, etc.] are to be evaluated [should be assessed] separately/independently. Further please note that any ‘statistical review’ is generally done under the assumption that (such) study specific methodological [as well as execution] issues are perfectly taken care of by the investigator(s). This review is not an exception to that and so does not cover clinical aspects {however, seldom comments are made only if those issues are intimately / scientifically related & intermingle with ‘statistical aspects’ of the study}. Agreed that ‘statistical methods’ are used as just tools here, however, they are vital part of methodology [and so should be given due importance]. I look at the manuscript in/with statistical view point, other reviewer(s) look(s) at it with different angle so that in totality the review is very comprehensive. However, there should be efforts from authors side to improve (may be by taking clues from reviewer’s comments). Therefore, please do not limit the revision only (with respect) to comments made here.**

**COMMENTS:** Although there are few appreciable points of this manuscript [example: application of Aligned Ranks Transform (ART) tests], I have few serious concerns {mainly regarding sample size and ‘Statistical analysis’ section}. First, refer to lines 128-129 where it is stated that “Based on an initial power calculation [24], a sample size of 68 participants was planned”, however, remember that since this is an independent publication [and not one in series], you are supposed to give some (more) details. Estimated number may be correct (but generally number per arm/group is reported/yielded). However, note that even quoted reference (24) says “it is planned that 80 mothers will be enrolled to anticipate possible participant withdrawal” but not mentioned “what is ‘anticipate possible participant withdrawal’ {i.e., drop-out} rate?” According to reference quoted, estimation of required sample size is based on sample of preterm (M=58.51, SD=12.57) and of term-born (M=65.9, SD=8.2) which implies that effect size assumed is much more than actually observed [refer to table-2]. Are the controls full term-born infants in the Neonatal Intensive Care Unit (NICU) environment? According to reference 24 (‘Control group’ section) ‘Participants in the control group will receive treatment as-usual’. That means they are also pre-term born infants in the Neonatal Intensive Care Unit (NICU) environment {which is very correct indeed}. What is true? The question (then) is, ‘are the assumptions made for estimation process, correct?’ Please give details of ‘control group’ selection & constitution. Can ‘control’ group definition be different for sample size estimation and for trial execution? Please mind you that this is a scientific/academic document and so all details should be clearly/correctly communicated (do not take readers’ for granted).

How the required minimum sample size for this study was determined is nevertheless a very-very important question [one of the important items in CONSORT checklist, item 7a] for any type of study (clinical trial or else) which needs to be discussed in adequate details {including assumptions made at the time of estimation, (expected) power of the study, software used, etc.}. In line 120 it is stated that “This manuscript follows the CONSORT guidelines.”. This implies that you know well that while reporting findings from [and even planning] ‘Clinical Trial’ one should follow CONSORT guidelines. Unfortunately, though it is said, even important items {like How sample size was determined (Item 7a), Random Sequence generation (Item 8a), Allocation concealment (Item 9), Blinding (Item 11a)} of/in CONSORT checklist are not found [since your article type is ‘Clinical Trial’, you are supposed to cover (with adequate detailing) these items in the report or even in ‘Protocol’]. Only stating (lines 376-8) that “a bias may have been introduced due to the unblinding of the participants and clinicians, as well as a possible contamination due to improvement of usual care by healthcare providers” is not sufficient. You have to give explanation as to why any sort of blinding was not possible / not done. For every required point, you are not supposed to indicate (just refer to) the protocol (though published). Mind you that this is an independent publication [and not one in series] as said earlier.

Note that according to table-2 on page 158 of Cohen’s paper “A power primer” in Psychological Bulletin, 1992, vol.:112, pp 155-159 [which is a sort of summary of the excellent book by Jacob Cohen entitled ‘Statistical power analysis for the behavioral sciences’, Academic Press, 1977, New York] even for medium effect size you need n=64 per group and for small effect size it is as large as 393 per group (type-I error=0.05, power=80%). Kindly note that ‘n’ is per group/arm & not for entire study/trial.

I request authors to read the following text [particularly in context of column 4 (P-values column) of Table 1 {‘Between-group differences at baseline - Maternal and infant sociodemographic variables’}] pasted from one famous standard textbook on ‘Medical Research Methodology’:

To provide a description of baseline characteristics is entirely reasonable (since it is clearly important in assessing to whom the results of the trial can be applied), however, statistical comparison of baseline characteristics when random allocation/assignment is used/done [often for good/standard/leading journals these days] is not required, because even if P-value(s) turn(s) out to be significant (while comparing baseline characteristics despite random allocation), it is, by definition, a false positive as you then are supposed to be testing ‘randomization’ then, which in any single trial may not balance all baseline characteristics (particularly when sample sizes are small). Remember that ‘randomization’ is a sort of ‘insurance’ and not a guarantee scheme. Authors may please refer to following articles:

References:

1. Stuart J. Pocock, et al., ‘Subgroup analysis, covariate adjustment and baseline comparisons in clinical trial reporting: current practice and problems’, Statistics in medicine, 2002; 21:2917–2930 [Particularly page 2927]
2. Harrington D, et al., ‘New guidelines for statistical reporting in the journal’, N Engl J Med 2019;381:285-6

[Important message (indirectly/ultimately indicated) from these articles: Never do any comparison with respect to ‘baseline’ characteristics {by applying statistical significance test(s)}, when allocation is done randomly].

However, Statistical comparison [only with respect to important/indicated variables] of baseline characteristics may be performed, to find out if analysis adjustment (say stratified analyses or else) is required with respect to these variables.

Note {referring to Table-1 with respect to Education; Migrant; Marital status; Number of children and Largo score categories}: There are few limitations/conditions [like more than 80% cells should have expected cell frequency more or equal to 5 as well as no cell frequency should be ‘zero’] of Chi-square test to keep in mind.

Though the measures/tools used are appropriate [examples: Primary outcome - Perceived Maternal Parenting Self-efficacy (PMP-SE); Secondary outcomes - Parental Stressor Scale : neonatal intensive care unit (F-PSS-NICU); Parenting Stress Index – Short Form (PSI - SF); Hospital Anxiety and Depression Scale (HADS); Edinburgh Postnatal Depression Scale (EPDS); etc.], most of them yield data that are in [at the most] ‘ordinal’ level of measurement [and not in ratio level of measurement for sure {as the score two times higher does not indicate presence of that parameter/phenomenon as double (for example, a Visual Analogue Scales VAS score or say ‘depression’ score)}]. This is given here because as in line 206-7 “We checked the equivalence between groups on social demographics and outcome measures at baseline using t-tests (and Wilcoxon rank sum tests)”, then the application of suitable non-parametric test(s) is/are indicated/advisable [even if distribution may be ‘Gaussian’ (also called ‘normal’)]. Agreed that there is/are no non-parametric test(s)/technique(s) available to be used as alternative in all situation(s) [suitable / most desired/applicable], but should be used whenever/wherever they are available. Good that at few places Wilcoxon rank sum tests are/were used (where?). However, in short use suitable non-parametric test(s)/technique(s) while dealing with data that are in ‘ordinal’ level of measurement even if distribution may be ‘Gaussian’.

By referring to lines 394-6 [Although this study did not find an effect of the intervention on perceived maternal self-efficacy, the experience of providing or receiving it was felt to be positive from the maternal side, as well as the multidisciplinary team.], I would like to point-out one fact. Remember that “Absence of evidence is not evidence of absence” [Altman DG, Bland JM. BMJ volume 311, 1995, p 485 (Reprinted: Australian Veterinary Journal 1996;74, 311)]. {Even when P-value is not significantly lower that is null hypothesis of no difference / no association is not rejected, (in short, result is not significant), that does not amount to evidence of absence i.e., it does not imply that there no difference / no association. It only implies that there is no (i.e., these samples do not provide) [say enough] evidence to prove (rather indicate with certain specified confidence level) the difference / association}. Therefore, conclusion(s) from any study [in which result(s) is/are not significant], should be drawn in the light of this fact. But would like to also note the second/later part that ‘it was felt to be positive from the maternal side, as well as the multidisciplinary team’. It may lead to doubt that “whether this ‘non-significance’ is resulted because of less than required sample size of the study [wrong control group used]?”

Moreover, limitations (if any) of the study are not mentioned/listed anywhere. Does that mean {according to authors} there are none? As pointed out in ‘important note’ above “This review pertains only to ‘statistical aspects’ of the study and so ‘clinical aspects’ should be assessed separately/independently [one should carefully consider/look at the clinical implications of the study]. In my opinion, to rescue this article (which is not quite impossible), some amount of re-vision (re-drafting) may be needed. However, please do not limit the revision only (with respect) to comments made here.
