# Supplementary material for: Joint observation in NICU (JOIN): A randomized controlled trial testing an early, one-session intervention during preterm care to improve perceived maternal self-efficacy and other mental health outcomes
Source: PLoS One. 2024 Apr 25;19(4):e0301594. doi: 10.1371/journal.pone.0301594 (PMC11045081; doi:10.1371/journal.pone.0301594)
Supplement: Attachment — Submitted filename: J.Schneider et al._Response to reviewers.docx [file pone.0301594.s006.docx]

Journal requirements:

When submitting your revision, we need you to address these additional requirements.

1. Please ensure that your manuscript meets PLOS ONE's style requirements, including those for file naming. The PLOS ONE style templates can be found at

<https://journals.plos.org/plosone/s/file?id=wjVg/PLOSOne_formatting_sample_main_body.pdf> and

<https://journals.plos.org/plosone/s/file?id=ba62/PLOSOne_formatting_sample_title_authors_affiliations.pdf>

*Response: The manuscript has been checked and changes have been made according to the journal’s style requirements.*

2. We note that you have indicated that data from this study are available upon request. PLOS only allows data to be available upon request if there are legal or ethical restrictions on sharing data publicly. For information on unacceptable data access restrictions, please see <http://journals.plos.org/plosone/s/data-availability#loc-unacceptable-data-access-restrictions>.

In your revised cover letter, please address the following prompts:

a) If there are ethical or legal restrictions on sharing a de-identified data set, please explain them in detail (e.g., data contain potentially identifying or sensitive patient information) and who has imposed them (e.g., an ethics committee). Please also provide contact information for a data access committee, ethics committee, or other institutional body to which data requests may be sent.

b) If there are no restrictions, please upload the minimal anonymized data set necessary to replicate your study findings as either Supporting Information files or to a stable, public repository and provide us with the relevant URLs, DOIs, or accession numbers. Please see <http://www.bmj.com/content/340/bmj.c181.long> for guidelines on how to de-identify and prepare clinical data for publication. For a list of acceptable repositories, please see <http://journals.plos.org/plosone/s/data-availability#loc-recommended-repositories>.

We will update your Data Availability statement on your behalf to reflect the information you provide.

*Response: The initial project was submitted to the ethics committee of the “Canton de Vaud” in December 2015. At that time, there was no requirement from the ethical board to mention the possibility of publicly sharing data in the information document to the participants. Thus, participants did not consent formally to share their data, even if de-identified. In that context, we are not allowed to publish or upload an anonymized data set in a repository. However, we could share the data upon reasonable request to the corresponding author. We mentioned this point in the cover letter.*

3. One of the noted authors is a group or consortium [JOIN Research Consortium]. In addition to naming the author group, please list the individual authors and affiliations within this group in the acknowledgments section of your manuscript. Please also indicate clearly a lead author for this group along with a contact email address.

*Response: The individual authors from the JOIN Research Consortium have been listed ant their affiliations checked. The lead author (Pr. Antje Horsch) has been identified in the acknowledgements section (Page 35, Line 515).*

4. Please upload a new copy of Figure 2 as the detail is not clear. Please follow the link for more information: <https://blogs.plos.org/plos/2019/06/looking-good-tips-for-creating-your-plos-figures-graphics/>" <https://blogs.plos.org/plos/2019/06/looking-good-tips-for-creating-your-plos-figures-graphics/>

*Response: A new version of the Figure 2 has been uploaded, with better resolution and higher font size of the legends. We used the PACE software to ensure our figure meets the requirements.*

5. Please review your reference list to ensure that it is complete and correct. If you have cited papers that have been retracted, please include the rationale for doing so in the manuscript text, or remove these references and replace them with relevant current references. Any changes to the reference list should be mentioned in the rebuttal letter that accompanies your revised manuscript. If you need to cite a retracted article, indicate the article’s retracted status in the References list and also include a citation and full reference for the retraction notice.

*Response: All the references have been checked.*


[Note: HTML markup is below. Please do not edit.]

Reviewers' comments:

Reviewer's Responses to Questions

**Comments to the Author**

1. Is the manuscript technically sound, and do the data support the conclusions?

The manuscript must describe a technically sound piece of scientific research with data that supports the conclusions. Experiments must have been conducted rigorously, with appropriate controls, replication, and sample sizes. The conclusions must be drawn appropriately based on the data presented.

Reviewer #1: Yes

Reviewer #2: Yes

2. Has the statistical analysis been performed appropriately and rigorously?

Reviewer #1: I Don't Know

Reviewer #2: I Don't Know

3. Have the authors made all data underlying the findings in their manuscript fully available?

The [PLOS Data policy](http://www.plosone.org/static/policies.action#sharing) requires authors to make all data underlying the findings described in their manuscript fully available without restriction, with rare exception (please refer to the Data Availability Statement in the manuscript PDF file). The data should be provided as part of the manuscript or its supporting information, or deposited to a public repository. For example, in addition to summary statistics, the data points behind means, medians and variance measures should be available. If there are restrictions on publicly sharing data—e.g. participant privacy or use of data from a third party—those must be specified.

Reviewer #1: Yes

Reviewer #2: Yes

4. Is the manuscript presented in an intelligible fashion and written in standard English?

PLOS ONE does not copyedit accepted manuscripts, so the language in submitted articles must be clear, correct, and unambiguous. Any typographical or grammatical errors should be corrected at revision, so please note any specific errors here.

Reviewer #1: Yes

Reviewer #2: Yes

5. Review Comments to the Author

Please use the space provided to explain your answers to the questions above. You may also include additional comments for the author, including concerns about dual publication, research ethics, or publication ethics. (Please upload your review as an attachment if it exceeds 20,000 characters)

Reviewer #1: Important note: This review pertains only to ‘statistical aspects’ of the study and so ‘clinical aspects’ [like medical importance, relevance of the study, ‘clinical significance and implication(s)’ of the whole study, etc.] are to be evaluated [should be assessed] separately/independently. Further please note that any ‘statistical review’ is generally done under the assumption that (such) study specific methodological [as well as execution] issues are perfectly taken care of by the investigator(s). This review is not an exception to that and so does not cover clinical aspects {however, seldom comments are made only if those issues are intimately / scientifically related & intermingle with ‘statistical aspects’ of the study}. Agreed that ‘statistical methods’ are used as just tools here, however, they are vital part of methodology [and so should be given due importance]. I look at the manuscript in/with statistical view point, other reviewer(s) look(s) at it with different angle so that in totality the review is very comprehensive. However, there should be efforts from authors side to improve (may be by taking clues from reviewer’s comments). Therefore, please do not limit the revision only (with respect) to comments made here.

*We thank the reviewer for his/her comments on the statistical part, which constitutes a very important component of the study. Issues regarding the clinical aspects have been addressed according to the comments of the second reviewer.*

*You will find below our point-to-point response to the comments. Please note that the page and line numbers to which the changes refer correspond to the clean version of the revised manuscript. We also added a separate bibliography with the references that were added or mentioned at the end of the present document.*

COMMENTS: Although there are few appreciable points of this manuscript [example: application of Aligned Ranks Transform (ART) tests], I have few serious concerns {mainly regarding sample size and ‘Statistical analysis’ section}. First, refer to lines 128-129 where it is stated that “Based on an initial power calculation [24], a sample size of 68 participants was planned”, however, remember that since this is an independent publication [and not one in series], you are supposed to give some (more) details. Estimated number may be correct (but generally number per arm/group is reported/yielded). However, note that even quoted reference (24) says “it is planned that 80 mothers will be enrolled to anticipate possible participant withdrawal” but not mentioned “what is ‘anticipate possible participant withdrawal’ {i.e., drop-out} rate?” According to reference quoted, estimation of required sample size is based on sample of preterm (M=58.51, SD=12.57) and of term-born (M=65.9, SD=8.2) which implies that effect size assumed is much more than actually observed [refer to table-2].

*Response: We agree with the reviewer that we should have better explained the power calculation in the present manuscript, and we must not take for granted the fact that the study protocol has been previously published. In that sense, we added a paragraph in the “Method” section detailing the sample size calculation, as follows (Pages 7-8, Lines 140-148) :* ***“The power calculation was based on previous publications measuring perceived parental self-efficacy in parents of term (Leahy-Warren, McCarthy, & Corcoran, 2012) and preterm (Barnes & Adamson-Macedo, 2007) neonates. As no previous study compared this specific outcome after a similar intervention in two groups of mothers of preterm neonates in the NICU, we made the assumption that the mothers of preterm neonates benefitting from the JOIN intervention will report comparable perceived self-efficacy as mothers of term neonates. Using the G*Power software (Faul, Erdfelder, Lang, & Buchner, 2007) allowing sample size determination and*** ***according to the published means and SD in these two samples (term: M=65.9, SD=8.2; preterm: M=58.1, SD 12.57), we needed to recruit 68 participants (α=0.05, 1-β=0.80, unilateral hypothesis). To anticipate possible withdrawal, we planned to enroll 80 mother-infant dyads in the present study”.***

*Secondarily, we acknowledge that the expected effect sizes were smaller than expected in the present study and we clarified this in the “Limitations” paragraph with this sentence (Page 32, Line 447-450) :* ***“Sixth, despite adequate sample size of the two groups based on the initial power calculation, we might also hypothesize that the absence of an effect of the intervention was related to the low number of participants, especially given that smaller effect sizes than expected were observed”.***

Are the controls full term-born infants in the Neonatal Intensive Care Unit (NICU) environment? According to reference 24 (‘Control group’ section) ‘Participants in the control group will receive treatment as-usual’. That means they are also pre-term born infants in the Neonatal Intensive Care Unit (NICU) environment {which is very correct indeed}. What is true? The question (then) is, ‘are the assumptions made for estimation process, correct?’ Please give details of ‘control group’ selection & constitution. Can ‘control’ group definition be different for sample size estimation and for trial execution? Please mind you that this is a scientific/academic document and so all details should be clearly/correctly communicated (do not take readers’ for granted).

*Response: We thank the reviewer for pointing this out and agree that there might a bit of confusion in the “Method” section regarding the control group. The control group comprises the same inclusion criteria as the intervention group, i.e. mothers of* ***preterm*** *neonates born between 28 and 32 6/7 weeks of gestational age. We acknowledge that the power calculation was based on previous publications on* ***term*** *neonates and that we made the assumption that mothers of preterm neonates in our control group might be comparable to mothers of term neonates in that study (Leahy-Warren et al., 2012) when reporting perceived self-efficacy. We did so because no previous study comparing perceived self-efficacy after a similar intervention as ours was available. Thus, we specified again in the “Trial design, procedure, data collection and timing” paragraph that both groups concerned mothers of preterm neonates, as follows (Page 8, Lines 150-153) :* ***“The*** *recruitment* ***of mothers of preterm neonates*** *was performed by research nurses* ***who approached the eligible participants once their infants were stable enough to avoid disturbance during a critical period and ensure their emotional availability”.***

*We also specified in the “Sample size” calculation paragraph the following sentence explaining the rationale of the power calculation (Page 7, Lines 141-144) : “****As no previous study compared this specific outcome after a similar intervention in two groups of mothers of preterm neonates in the NICU,*** *we assumed that the mothers of preterm neonates benefitting from the JOIN intervention will report comparable perceived self-efficacy as mothers of term neonates”.*

How the required minimum sample size for this study was determined is nevertheless a very-very important question [one of the important items in CONSORT checklist, item 7a] for any type of study (clinical trial or else) which needs to be discussed in adequate details {including assumptions made at the time of estimation, (expected) power of the study, software used, etc.}. In line 120 it is stated that “This manuscript follows the CONSORT guidelines.”. This implies that you know well that while reporting findings from [and even planning] ‘Clinical Trial’ one should follow CONSORT guidelines. Unfortunately, though it is said, even important items {like How sample size was determined (Item 7a), Random Sequence generation (Item 8a), Allocation concealment (Item 9), Blinding (Item 11a)} of/in CONSORT checklist are not found [since your article type is ‘Clinical Trial’, you are supposed to cover (with adequate detailing) these items in the report or even in ‘Protocol’]. Only stating (lines 376-8) that “a bias may have been introduced due to the unblinding of the participants and clinicians, as well as a possible contamination due to improvement of usual care by healthcare providers” is not sufficient. You have to give explanation as to why any sort of blinding was not possible / not done. For every required point, you are not supposed to indicate (just refer to) the protocol (though published). Mind you that this is an independent publication [and not one in series] as said earlier.

*Response: We agree with the reviewer that we referred a lot to our previous publication of the study protocol and that the present manuscript lacks details, especially regarding the CONSORT guidelines requirements. We completed the “Sample size calculation” paragraph by specifying the type of software we used for the sample size determination, as follows (Pages 7-8, Lines 144-147) :* **“*Using the G*Power software(Faul et al., 2007) allowing sample size determination and according to the published means and SD in these two samples (term: M=65.9, SD=8.2; preterm: M=58.1, SD 12.57), we needed to recruit 68 participants (α=0.05, 1-β=0.80, unilateral hypothesis)”.***

*We also made changes accordingly in the* *“Trial design, procedure, data collection and timing” paragraph by adding information on random sequence generation, allocation concealment, blinding and procedures in each group. Modifications were the following (Pages 8-9, Lines 150-172) : “This monocentric RCT aimed to test an intervention compared with treatment-as-usual. The recruitment of* ***mothers of preterm neonates*** *was performed by research nurses* ***who approached the eligible participants once their infants were stable enough to avoid disturbance during a critical period and ensure their emotional availability. Using a computer-generated list of random blocks (***[***https://www.sealedenvelope.com***](https://www.sealedenvelope.com)***)****, a research assistant generated the random allocation sequence,* ***which was concealed from the principal investigator in sequentially numbered, opaque, and sealed envelopes.*** *Two groups of participants were created on the basis of a randomisation 1:1.* ***The envelopes were opened after the signature of the consent and the completion of the baseline questionnaires (for further details see (Schneider et al., 2019)).***

***The principal investigator and the statistician were blinded to group allocation. As the intervention took place in the clinical context of the NICU and no placebo intervention was performed, the clinical team administering the intervention was de facto not blinded for group allocation but did not participate in subsequent data collection and follow-up. Similarly, no blinding was possible for the participants.***

***Control group***

*After giving written consent, participants of the* ***control*** *group were asked to complete a set of questionnaires at three time points: T1 at baseline (recruitment), T2 at 1-month after enrollment, and T3 at 6 months corrected-age of the infant (CA).* ***The mother-infant dyads received treatment-as-usual in the NICU after a preterm birth.***

***Intervention group***

***Mothers assigned to the intervention group were asked to complete the questionnaires at the three time points defined previously. The intervention was performed after enrollment and completion of the baseline questionnaires once the infant’s clinical state was stabilized”.***

Note that according to table-2 on page 158 of Cohen’s paper “A power primer” in Psychological Bulletin, 1992, vol.:112, pp 155-159 [which is a sort of summary of the excellent book by Jacob Cohen entitled ‘Statistical power analysis for the behavioral sciences’, Academic Press, 1977, New York] even for medium effect size you need n=64 per group and for small effect size it is as large as 393 per group (type-I error=0.05, power=80%). Kindly note that ‘n’ is per group/arm & not for entire study/trial.

*Response: We thank the reviewer for this comment. We agreed that this information was missing, and we added the number of participants per group as well as the estimated effect size / statistical power in tables 2 and 3.*

I request authors to read the following text [particularly in context of column 4 (P-values column) of Table 1 {‘Between-group differences at baseline - Maternal and infant sociodemographic variables’}] pasted from one famous standard textbook on ‘Medical Research Methodology’:
To provide a description of baseline characteristics is entirely reasonable (since it is clearly important in assessing to whom the results of the trial can be applied), however, statistical comparison of baseline characteristics when random allocation/assignment is used/done [often for good/standard/leading journals these days] is not required, because even if P-value(s) turn(s) out to be significant (while comparing baseline characteristics despite random allocation), it is, by definition, a false positive as you then are supposed to be testing ‘randomization’ then, which in any single trial may not balance all baseline characteristics (particularly when sample sizes are small). Remember that ‘randomization’ is a sort of ‘insurance’ and not a guarantee scheme.

Authors may please refer to following articles:
References:
1. Stuart J. Pocock, et al., ‘Subgroup analysis, covariate adjustment and baseline comparisons in clinical trial reporting: current practice and problems’, Statistics in medicine, 2002; 21:2917–2930 [Particularly page 2927]
2. Harrington D, et al., ‘New guidelines for statistical reporting in the journal’, N Engl J Med 2019;381:285-6
[Important message (indirectly/ultimately indicated) from these articles: Never do any comparison with respect to ‘baseline’ characteristics {by applying statistical significance test(s)e, when allocation is done randomly].
However, Statistical comparison [only with respect to important/indicated variables] of baseline characteristics may be performed, to find out if analysis adjustment (say stratified analyses or else) is required with respect to these variables.

Note {referring to Table-1 with respect to Education; Migrant; Marital status; Number of children and Largo score categoriese: There are few limitations/conditions [like more than 80% cells should have expected cell frequency more or equal to 5 as well as no cell frequency should be ‘zero’] of Chi-square test to keep in mind.

*Response: Thank you for very much for your comment. We understand that the statistical comparison of baseline characteristics could be misleading when allocation is done randomly. We removed this information from table 1, as well as the related text describing the type of statistical tests carried out.*

Though the measures/tools used are appropriate [examples: Primary outcome - Perceived Maternal Parenting Self-efficacy (PMP-SE); Secondary outcomes - Parental Stressor Scale : neonatal intensive care unit (F-PSS-NICU); Parenting Stress Index – Short Form (PSI - SF); Hospital Anxiety and Depression Scale (HADS); Edinburgh Postnatal Depression Scale (EPDS); etc.], most of them yield data that are in [at the most] ‘ordinal’ level of measurement [and not in ratio level of measurement for sure {as the score two times higher does not indicate presence of that parameter/phenomenon as double (for example, a Visual Analogue Scales VAS score or say ‘depression’ score)}Res]. This is given here because as in line 206-7 “We checked the equivalence between groups on social demographics and outcome measures at baseline using t-tests (and Wilcoxon rank sum tests)”, then the application of suitable non-parametric test(s) is/are indicated/advisable [even if distribution may be ‘Gaussian’ (also called ‘normal’)]. Agreed that there is/are no non-parametric test(s)/technique(s) available to be used as alternative in all situation(s) [suitable / most desired/applicable], but should be used whenever/wherever they are available. Good that at few places Wilcoxon rank sum tests are/were used (where?). However, in short use suitable non-parametric test(s)/technique(s) while dealing with data that are in ‘ordinal’ level of measurement even if distribution may be ‘Gaussian’.

*Response: We thank the reviewer for this relevant comment. Due to the lack of space, we provided the minimum explanation required to figure out the statistical analyses taken. We understand that the information provided could have been a bit too scarce so, let us take the opportunity to further detail the approach. To analyze the between-group differences at post intervention and 6 months, the selected covariates as well as the outcomes baselines were introduced in ANCOVA models, at post intervention and 6 months respectively and the corresponding effect sizes were expressed as eta squared. We split the 2x3 factorial design into two simple within-subject (repeated measures) design to analyze the within-group differences at pre-intervention, post-intervention, and 6 months. As the outcomes scores (aggregated from Likert scales items) where somewhat skewed, we used non-parametric tests when possible. When there was no covariate statistically significantly related with the outcome, we used ART model and LME models otherwise. For each group, the effect size (partial eta squared) and related statistical power were estimated at pre-intervention, post-intervention, and 6-month time points. The “Statistical analysis” section was has been modified accordingly, as follows (Pages 12-13, Lines 249-269) : “All analyses were carried out with RStudio v1.3.1093 running R v4.1.0. We disregarded participants having not replied to any items in questionnaires (S1 Table). We imputed* ***questionnaires*** *missing* ***at random*** *values per time point and at item level using the* ***median*** *value of the* ***participant items****. Four participants were not assigned to a group and thus excluded from the analysis. The flow chart of the study is available in Fig 1.*

***Fig 1. Flow chart of the study***

***As the outcomes, resulting from the aggregation of Likert scale type of items, were sometime deviating from normal distributions we tried to enforce non-parametric tests where possible. To analyze the between-group differences at post intervention and 6 months, outcomes baselines as well as the potential confounding variables were included in covariance analyses, where applicable. These included the age of the mother, the gender of the baby, the socio-economic status (Largo et al., 1989), and number of children. For the primary outcome analyses, the mMOS-SS score at baseline was included in the covariance analysis as well, as this correlated statistically significantly with the primary outcome at baseline. The effect sizes and the corresponding statistical power were estimated at post intervention and 6 months.***

***We split the 2x3 factorial design into two simple within-subject (repeated measures) designs to analyze the within-group differences at pre-intervention, post-intervention, and 6 months. To consider the repeated measure structure we used Aligned Ranks Transform (ART) models when there was no covariate statistically significantly related with the outcome and Linear Mixed Effect (LME) models otherwise. For each group, the effect size and related statistical power were estimated at pre-intervention, post-intervention, and 6-month”.***

*As you rightly mentioned, some parametric tests do not have a non-parametric counterpart and therefore are difficult to avoid, even though the analyzed sample distributions somewhat deviate from normality. We were aware that the sample outcomes’ distributions were not always normally distributed and that the corresponding underlying populations were probably not normally distributed either, but the tests were applied on the questionnaires scores, not directly on their Likert scale type of items. To validate our approach, we also relied on studies having shown that parametric tests not only could be used with ordinal data / Likert scales, but also are generally more robust than non-parametric tests (Norman, 2010). Finally, since the tests residuals were rather normally distributed, we were confident that the results yielded from that first approach could be correctly interpreted (i.e., none of the covariates’ effects were significant on the outcomes).*

By referring to lines 394-6 [Although this study did not find an effect of the intervention on perceived maternal self-efficacy, the experience of providing or receiving it was felt to be positive from the maternal side, as well as the multidisciplinary team.], I would like to point-out one fact. Remember that “Absence of evidence is not evidence of absence” [Altman DG, Bland JM. BMJ volume 311, 1995, p 485 (Reprinted: Australian Veterinary Journal 1996;74, 311)]. {Even when P-value is not significantly lower that is null hypothesis of no difference / no association is not rejected, (in short, result is not significant), that does not amount to evidence of absence i.e., it does not imply that there no difference / no association. It only implies that there is no (i.e., these samples do not provide) [say enough] evidence to prove (rather indicate with certain specified confidence level) the difference / association}. Therefore, conclusion(s) from any study [in which result(s) is/are not significant], should be drawn in the light of this fact. But would like to also note the second/later part that ‘it was felt to be positive from the maternal side, as well as the multidisciplinary team’. It may lead to doubt that “whether this ‘non-significance’ is resulted because of less than required sample size of the study [wrong control group used]?”

*Response: We thank the reviewer for this comment and agree that even though no effect of the intervention has been found on perceived self-efficacy as reported by mothers of preterm infants, it does not mean that it does not definitely exist. Also, we acknowledge that, given that the sample size calculation has been made based on previously published means and SD related to perceived self-efficacy in a population of term and preterm neonates, it might not be completely adequate and that a larger sample size may have brought significant results. Thus, we added these elements in the “Discussion” section of the manuscript as follows : (Page 32, Lines 447-452) “****Sixth, despite adequate sample size of the two groups based on the initial power calculation, we might also hypothesize that the absence of an effect of the intervention was related to the low number of participants, especially given that smaller effect sizes than expected were observed. The sample size calculation was performed on previous published means related to perceived parental self-efficacy in a population of mothers of term (Leahy-Warren et al., 2012) and preterm (Barnes & Adamson-Macedo, 2007) neonates, which might prove not to have been entirely suitable. […]*** *(Page 33, Lines 470-473) Although this study did not find an effect of the intervention on perceived maternal self-efficacy,* ***it does not mean that no association would have been found in a different context given the limitations that have been raised above, especially the sample size, the choice of a mildly vulnerable population or the single intervention design”.***

Moreover, limitations (if any) of the study are not mentioned/listed anywhere. Does that mean {according to authors} there are none? As pointed out in ‘important note’ above “This review pertains only to ‘statistical aspects’ of the study and so ‘clinical aspects’ should be assessed separately/independently [one should carefully consider/look at the clinical implications of the study]. In my opinion, to rescue this article (which is not quite impossible), some amount of re-vision (re-drafting) may be needed. However, please do not limit the revision only (with respect) to comments made here.

*Response: We agree with the reviewer that no “Limitations” paragraph per se appeared in the “Discussion” section of the manuscript. In fact, the paragraph where the possible reasons for not observing group differences were discussed, was actually detailing the limitations of the study. We made it more visible by adding an header “Limitations” to the paragraph and by highlighting it in the paragraph as follows (Page 31, Lines 416-419) : “Possible reasons,* ***which might be regarded as limitations of the study****, are related to 1) the infants’ and mothers’ sociodemographic characteristics, 2) the high standard of routine care, 3) the study design, 4) the intervention providers,* ***5) the choice of the primary outcome, and 6) the sample size”.***

*As such, we also completed the list of the limitations by adding the possible limitation represented by the sample size as discussed in the response to the comment above.*

Reviewer #2: This is a very well written paper on an important subject. NICU parents and infants are a vulnerable population and it is critical to develop interventions that assist them in establishing more optimal relationships to support infant development and parental mental health. Ideal interventions are feasible to disseminate in NICUs with varying degrees of psychosocial support and can provide support for parents and infants from varying backgrounds. JOIN is possibly one such intervention, and therefore, research into its effectiveness in improving parental self-efficacy and parental and infant mental health and developmental outcomes is very important. The authors designed this RCT based on previous studies on JOIN and used self-report questionnaires to assess multiple important outcomes. Ultimately, they do not find a meaningful difference between the two arms of the study, but they go on to describe potential reasons for this lack of a difference in a very thoughtful way. Overall, I believe this is an important research in the field and lays the foundation for future inquiries into this intervention.

*We thank the reviewer for his/her positive comments and for taking the time to review our manuscript. You will find below our point-to-point response to the comments. Please note that the page and line numbers to which the changes refer correspond to the clean version of the revised manuscript. We also added a separate bibliography with the references that were added or mentioned at the end of the present document.*

Here are a few minor questions/suggested revisions:

Abstract:

- line 25: what do they mean by "adjusted parent-infant relationship", consider a different adjective.

*Response: We meant by “adjusted parent-infant relationship” that the parents adapt their behavior to the needs of their infant and that the infant reacts accordingly to what is provided to him/her in an interactive dialogue. We agree that this cannot be correctly summarized by the term “adjusted” and we modified it by “****adaptive parent-infant relationship****” (Page 3, Lines 29-30).*

Introduction:

- lines 82-86: Please further elaborate on how parental self-efficacy is distinguished from confidence or competence.

*Response: We better defined the concept of self-efficacy to help distinguishing it from confidence and competence, as summarized by (Montigny & Lacharite, 2005). We added this sentence (Page 5, Lines 87-93): “****The latter refers to the conviction that others hold about the parent’s abilities to achieve something. In that sense, the parents may possess the skills but are not necessarily able to integrate them in a specific task. On the other hand, self-confidence refers to the belief in one’s own abilities and judgements and constitutes a stable state of certainty which is not situation-dependent. While relying on self-confidence, self-efficacy more precisely relates to the conviction to succeed in actively learning new skills and accomplishing specific tasks*** ***(e.g., the mother’s ability to feed or soothe her baby), while adapting to unfamiliar situations (Montigny & Lacharite, 2005)****”.*

- line 106: instead of "by the intervention", consider "In this intervention"

*Response: We modified the text according to your proposal (Page 6. Line 115).*

Methods"

-line 118: instead of the "protocol of the", consider "the protocol for the"

*Response: We made the change according to the suggestion (Page 7, Line 127).*

- it would be helpful to more clearly divide the exclusion criteria to maternal factors and infant factors. Consider putting ":" after each rather than ";".

*Response: The changes have been made accordingly (Page 7, Lines 133-137). “Exclusion criteria* ***regarding the mother*** *were: age less than 18 years, established intellectual disability or psychotic illness, insufficient local language skills level to complete questionnaires.* ***Exclusion criteria regarding the preterm neonate was related to*** *cardiorespiratory instability (severe brady-apnoea syndrome, oxygen requirement >30%) to ensure survival during the study period”.*

-line 125: instead of "aged", consider "age"

*Response: We modified the sentence according to the suggestion (Page 7, Line 134).*

-line 141: instead of "this moment", consider "this period"

*Response: We thank the reviewer for the suggestion and made the change (Page 9, Line 176).*

-line 163: what aspect of the infant?

*Response: We described in more details to what referred “aspect of the infant” to, as follows (Page 10, Lines 199-202) : “This 31 item-questionnaire with good psychometric properties assesses the parental stress specifically in NICU in three domains: impact of the environment, behaviour and aspect* ***(e.g., respiratory pattern, colour, equipment, skin lesions)*** *of the infant, and parental role.*

- in the abstract it is mentioned that authors measure PTSD outcomes as well, but the included measurements only look at stress, anxiety and depression, please clarify this.

*Response: We thank the reviewer for pointing it out. Initially, the study protocol included the ‘Posttraumatic Diagnosis Scale’ questionnaire. However, due to technical problems, answers to this questionnaire were incomplete and we had to discard it from the results. Thus, we completely agree with the reviewer that we did not measure specifically PTSD outcomes in the present study. We removed the mention of “post-traumatic stress” in the “Abstract” and in the “Introduction” section – secondary objectives.*

- for all the outcome measures used, please consider commenting on their psychosocial properties for the NICU population

*Response:*

*We thank the reviewer for giving us the opportunity to complete the description of the questionnaires that you will find below.*

*Perceived Maternal Parenting Self-efficacy (PMP-SE):*

*This questionnaire was specifically developed for mothers of preterm neonates, but for a generally healthy population and in a specific country. We added this information in the text as follows (Page 9, Lines 186-191) :* “*This 20-items questionnaire includes four subscales (care taking procedure, evoking behaviours, reading behaviours/signalling, situational beliefs) specifically designed to assess perception of parental ability for mothers* ***experiencing NICU hospitalization of their preterm neonates. The questionnaire was developed in a population of relatively healthy preterm neonates and authors warn of the need of a cross-cultural validation for a more general application outside Europe”.***

*Parental Stressor Scale : neonatal intensive care unit (F-PSS-NICU)*

*The F-PSS-NICU was ‘****designed to measure parental perception of stressors arising from the physical and psychosocial environment of the NICU****’ as specified in the original publication. We added details in the main text (Page 10, Line 202-203).*

*Parenting Stress Index – Short Form (PSI - SF):*

*We added information based on a previous publication reporting results from parents of a preterm population using this questionnaire as follows (Page 10, Lines 209-212) : “This questionnaire has been validated in French and displays good psychometric properties in studies involving parents of children born preterm* ***with various degree of prematurity and medical risk and issued from a racially and socio-economically diverse population****”.*

*Hospital Anxiety and Depression Scale (HADS)*

*This questionnaire was not specifically developed to assess mental health of parents of preterm neonates in the NICU. However, a recent study reported interesting findings on a population of parents after a very preterm birth with diverse background. Identified risk factors for anxiety and depression were length of the hospital stay, migration background, educational level, and employment status. We added the following sentence in the text (Pages 10-11, Lines 216-219) : “****While the original questionnaire was not developed to specifically assess mental health of parents after preterm birth, (Vriend et al., 2021) recently reported increased level of anxiety and depression in parents of preterm neonates with social risks (migration background, educational level and employment status****)”.*

*Edinburgh Postnatal Depression Scale (EPDS)*

*The original version of this questionnaire was developed on a general population of mothers. Several publications reported outcomes in a population of preterm infants that were hospitalized in a NICU. Thus. We added the following sentence to the text with references (Page 11, Lines 224-226) :* ***“Although this questionnaire does not assess depressive symptoms specifically after a preterm birth, multiple*** *studies reported these outcomes in a population of mothers of preterm infants (Kleine et al., 2022; Mautner et al., 2021).”.*

*Mother-to-Infant Bonding Scale (MIBS)*

*Few studies used this questionnaire to specifically assess mother-infant bonding after NICU hospitalization. However, our group published a study in 2017 examining parental mental health after neonatal asphyxia. We completed the paragraph with this sentence and reference (Page 11, Lines 230-232) : “****The questionnaire does not specifically assess bonding after NICU hospitalization, but our group reported this outcome in mothers of term neonates admitted after neonatal asphyxia (Horsch et al., 2017)”.***

*Infant Behaviour Questionnaire–Revised Very Short Form (IBQ-R)*

*This general questionnaire exploring the temperament of children during the first year of age has previously been used in a population of preterm children. We added this mention in the paragraph as follows (Page 11, Lines 237-239) : “****This questionnaire previously proved to reliably assess temperament in former preterm children during the first year of age (Vaccaro et al., 2021)”.***

Discussion:

The discussion is particularly strong and well written. It would be helpful if authors explain why they chose a one session intervention (question of the dosing of the intervention) and briefly tell us how they envision adjusting the intervention to each unique NICU dyad's needs.

*Response: We acknowledge with the reviewer that the considerations regarding the choice of the intervention design were not clearly stated.* *The JOIN intervention was based on a previous study from part of our group (Borghini et al., 2014) demonstrating a positive effect of a 3-step intervention program including the JOIN observation on posttraumatic stress symptoms and quality of mother-infant interactions. In addition, the intervention was based on four theorical models of infant development as presented in the “Introduction” section. This has been better explained in the “Discussion” section as follows (Pages 30-31, Lines 403-412) : “**Nevertheless, the JOIN observation, as part of an intervention program, had in a previous study demonstrated to have an effect on maternal and child outcomes in our centre, including lower maternal posttraumatic stress symptoms and increased sensitivity, as well as improved infant cooperation during interactions at 4 months of age (Borghini et al., 2014).* ***In addition, the JOIN observation combines elements originating from four distinct theories of infant development, namely the method described by Brazelton et Nugent (Brazelton & Nugent, 1995), the synactive model of Als (Als & Pediatrics, 1986), the sensori-motor approach of Bullinger (Bullinger & Goubet, 1999) and the interactive guidance (Kennedy, Ball, & Barlow, 2017; Rusconi-Serpa, Sancho Rossignol, & McDonough, 2009). Given that there was evidence for each element to promote either neonatal support or parental sensitivity, we were reasonably confident that incorporating them to build this*** *early, one-session intervention in the NICU* ***would be effective.”***

*In the “Perspective” paragraph, we better defined how we could adapt the intervention to the parent-infant needs as follows: (Page 34, Lines 485-489) “Identifying these parents could be based on screening parents regarding their psychological vulnerability or distress whilst their infant is still hospitalised in the NICU,* ***for instance by using specific questionnaires, such as the m-MOS-SS, EPDS or HADS.*** ***Similarly, the sickest infants with neonatal complications bringing vital risks should be identified, as their parents may experience more mental health difficulties related to this critical situation.*** ***[…]*** *(Page 34, Lines 492-497)* ***Hence, previous interventions that demonstrated positive effects on mother-infant outcomes provided more sustained support, at least for a 4-week period (Hane et al., 2015; Melnyk et al., 2006) or at several time-points from term equivalent age to 4 months of corrected age, while the infant has already been discharged (Borghini et al., 2014). More individualized parenting support adapted to the parental and infant needs in terms of duration, frequency and content might be developed too, focusing on the more vulnerable population”.***

Their discussion about the role of sociodemographic factors in their population (high education level, etc), less medical acuity, possibility of directly measuring mother's interactive behaviors rather than their self report of their self-efficacy, and the strength of their treatment as usual (good developmental care program and strong resources for psychological support on the unit) are all very well thought out.

I very much enjoyed this paper and look forward to seeing further research on JOIN!

6. PLOS authors have the option to publish the peer review history of their article ([what does this mean?](https://journals.plos.org/plosone/s/editorial-and-peer-review-process#loc-peer-review-history)). If published, this will include your full peer review and any attached files.


If you choose “no”, your identity will remain anonymous but your review may still be made public.


**Do you want your identity to be public for this peer review?** For information about this choice, including consent withdrawal, please see our [Privacy Policy](https://www.plos.org/privacy-policy).

Reviewer #1: No

Reviewer #2: No

[NOTE: If reviewer comments were submitted as an attachment file, they will be attached to this email and accessible via the submission site. Please log into your account, locate the manuscript record, and check for the action link "View Attachments". If this link does not appear, there are no attachment files.]

While revising your submission, please upload your figure files to the Preflight Analysis and Conversion Engine (PACE) digital diagnostic tool, <https://pacev2.apexcovantage.com/>. PACE helps ensure that figures meet PLOS requirements. To use PACE, you must first register as a user. Registration is free. Then, login and navigate to the UPLOAD tab, where you will find detailed instructions on how to use the tool. If you encounter any issues or have any questions when using PACE, please email PLOS at [figures@plos.org](mailto:figures@plos.org). Please note that Supporting Information files do not need this step.

***References :***

*Als, H. J. P., & Pediatrics, O. T. i. (1986). A synactive model of neonatal behavioral organization: framework for the assessment of neurobehavioral development in the premature infant and for support of infants and parents in the neonatal intensive care environment. 6(3-4), 3-53.*

*Barnes, C. R., & Adamson-Macedo, E. N. (2007). Perceived Maternal Parenting Self-Efficacy (PMP S-E) tool: development and validation with mothers of hospitalized preterm neonates. J Adv Nurs, 60(5), 550-560. doi:10.1111/j.1365-2648.2007.04445.x*

*Borghini, A., Habersaat, S., Forcada-Guex, M., Nessi, J., Pierrehumbert, B., Ansermet, F., & Muller-Nix, C. (2014). Effects of an early intervention on maternal post-traumatic stress symptoms and the quality of mother-infant interaction: the case of preterm birth. Infant Behav Dev, 37(4), 624-631. doi:10.1016/j.infbeh.2014.08.003*

*Brazelton, T. B., & Nugent, J. K. (1995). Neonatal behavioral assessment scale: Cambridge University Press.*

*Bullinger, A., & Goubet, N. J. E. (1999). Le bébé prématuré, acteur de son développement. 52(1), 27-32.*

*Faul, F., Erdfelder, E., Lang, A. G., & Buchner, A. (2007). G*Power 3: a flexible statistical power analysis program for the social, behavioral, and biomedical sciences. Behav Res Methods, 39(2), 175-191. doi:10.3758/bf03193146*

*Hane, A. A., Myers, M. M., Hofer, M. A., Ludwig, R. J., Halperin, M. S., Austin, J., . . . Welch, M. G. (2015). Family nurture intervention improves the quality of maternal caregiving in the neonatal intensive care unit: evidence from a randomized controlled trial. J Dev Behav Pediatr, 36(3), 188-196. doi:10.1097/DBP.0000000000000148*

*Horsch, A., Jacobs, I., Gilbert, L., Favrod, C., Schneider, J., Morisod Harari, M., & Bickle Graz, M. (2017). Impact of perinatal asphyxia on parental mental health and bonding with the infant: a questionnaire survey of Swiss parents. BMJ Paediatr Open, 1(1), e000059. doi:10.1136/bmjpo-2017-000059*

*Kennedy, H., Ball, K., & Barlow, J. (2017). How does video interaction guidance contribute to infant and parental mental health and well-being? Clin Child Psychol Psychiatry, 22(3), 500-517. doi:10.1177/1359104517704026*

*Kleine, I., Vamvakas, G., Lautarescu, A., Falconer, S., Chew, A., Counsell, S., . . . Nosarti, C. (2022). Postnatal maternal depressive symptoms and behavioural outcomes in term-born and preterm-born toddlers: a longitudinal UK community cohort study. BMJ Open, 12(9), e058540. doi:10.1136/bmjopen-2021-058540*

*Largo, R. H., Pfister, D., Molinari, L., Kundu, S., Lipp, A., & Duc, G. (1989). Significance of prenatal, perinatal and postnatal factors in the development of AGA preterm infants at five to seven years. Dev Med Child Neurol, 31(4), 440-456. Retrieved from* [*https://www.ncbi.nlm.nih.gov/pubmed/2680687*](https://www.ncbi.nlm.nih.gov/pubmed/2680687)

[*https://onlinelibrary.wiley.com/doi/abs/10.1111/j.1469-8749.1989.tb04022.x?sid=nlm%3Apubmed*](https://onlinelibrary.wiley.com/doi/abs/10.1111/j.1469-8749.1989.tb04022.x?sid=nlm%3Apubmed)

*Leahy-Warren, P., McCarthy, G., & Corcoran, P. (2012). First-time mothers: social support, maternal parental self-efficacy and postnatal depression. J Clin Nurs, 21(3-4), 388-397. doi:10.1111/j.1365-2702.2011.03701.x*

*Mautner, E., Stern, C., Avian, A., Deutsch, M., Scholl, W., & Greimel, E. (2021). Neonates in the Intensive Care Unit: Maternal Health-Related Quality of Life and Depression After Term and Preterm Births. Front Pediatr, 9, 684576. doi:10.3389/fped.2021.684576*

*Melnyk, B. M., Feinstein, N. F., Alpert-Gillis, L., Fairbanks, E., Crean, H. F., Sinkin, R. A., . . . Gross, S. J. (2006). Reducing premature infants' length of stay and improving parents' mental health outcomes with the Creating Opportunities for Parent Empowerment (COPE) neonatal intensive care unit program: a randomized, controlled trial. Pediatrics, 118(5), e1414-1427. doi:10.1542/peds.2005-2580*

*Montigny, F., & Lacharite, C. (2005). Perceived parental efficacy: concept analysis. J Adv Nurs, 49(4), 387-396. doi:10.1111/j.1365-2648.2004.03302.x*

*Norman, G. (2010). Likert scales, levels of measurement and the "laws" of statistics. Adv Health Sci Educ Theory Pract, 15(5), 625-632. doi:10.1007/s10459-010-9222-y*

*Rusconi-Serpa, S., Sancho Rossignol, A., & McDonough, S. C. (2009). Video feedback in parent-infant treatments. Child Adolesc Psychiatr Clin N Am, 18(3), 735-751. doi:10.1016/j.chc.2009.02.009*

*Schneider, J., Borghini, A., Morisod Harari, M., Faure, N., Tenthorey, C., Le Berre, A., . . . Consortium, J. R. (2019). Joint observation in NICU (JOIN): study protocol of a clinical randomised controlled trial examining an early intervention during preterm care. BMJ Open, 9(3), e026484. doi:10.1136/bmjopen-2018-026484*

*Vaccaro, S. M., Tofighi, D., Moss, N., Rieger, R., Lowe, J. R., Phillips, J., & Erickson, S. J. (2021). The association of infant temperament and maternal sensitivity in preterm and full-term infants. Infant Ment Health J, 42(3), 374-385. doi:10.1002/imhj.21915*

*Vriend, E., Leemhuis, A., Flierman, M., van Schie, P., Nollet, F., & Jeukens-Visser, M. (2021). Mental health monitoring in parents after very preterm birth. Acta Paediatr, 110(11), 2984-2993. doi:10.1111/apa.16064*
