# Supplementary material for: Joint observation in NICU (JOIN): A randomized controlled trial testing an early, one-session intervention during preterm care to improve perceived maternal self-efficacy and other mental health outcomes
Source: PLoS One. 2024 Apr 25;19(4):e0301594. doi: 10.1371/journal.pone.0301594 (PMC11045081; doi:10.1371/journal.pone.0301594)
Supplement: Attachment — Submitted filename: J.Schneider et al._Response to reviewers_2nd revision.docx [file pone.0301594.s007.docx]

# **Response to reviewers**

Journal Requirements:

Please review your reference list to ensure that it is complete and correct. If you have cited papers that have been retracted, please include the rationale for doing so in the manuscript text, or remove these references and replace them with relevant current references. Any changes to the reference list should be mentioned in the rebuttal letter that accompanies your revised manuscript. If you need to cite a retracted article, indicate the article’s retracted status in the References list and also include a citation and full reference for the retraction notice.

*Response: The reference list has been checked, according to the Journal requirements.*

Additional Editor Comments:

I would like to suggest the authors to address the concerns raised by reviewer 1.

*Response: Please see below our response to the comments raised by the reviewer 1.*

[Note: HTML markup is below. Please do not edit.]

Reviewers' comments:

Reviewer's Responses to Questions

**Comments to the Author**

1. If the authors have adequately addressed your comments raised in a previous round of review and you feel that this manuscript is now acceptable for publication, you may indicate that here to bypass the “Comments to the Author” section, enter your conflict of interest statement in the “Confidential to Editor” section, and submit your "Accept" recommendation.

Reviewer #1: (No Response)

Reviewer #2: All comments have been addressed

2. Is the manuscript technically sound, and do the data support the conclusions?

The manuscript must describe a technically sound piece of scientific research with data that supports the conclusions. Experiments must have been conducted rigorously, with appropriate controls, replication, and sample sizes. The conclusions must be drawn appropriately based on the data presented.

Reviewer #1: (No Response)

Reviewer #2: Yes

3. Has the statistical analysis been performed appropriately and rigorously?

Reviewer #1: (No Response)

Reviewer #2: I Don't Know

4. Have the authors made all data underlying the findings in their manuscript fully available?

The [PLOS Data policy](http://www.plosone.org/static/policies.action#sharing) requires authors to make all data underlying the findings described in their manuscript fully available without restriction, with rare exception (please refer to the Data Availability Statement in the manuscript PDF file). The data should be provided as part of the manuscript or its supporting information, or deposited to a public repository. For example, in addition to summary statistics, the data points behind means, medians and variance measures should be available. If there are restrictions on publicly sharing data—e.g. participant privacy or use of data from a third party—those must be specified.

Reviewer #1: (No Response)

Reviewer #2: No

5. Is the manuscript presented in an intelligible fashion and written in standard English?

PLOS ONE does not copyedit accepted manuscripts, so the language in submitted articles must be clear, correct, and unambiguous. Any typographical or grammatical errors should be corrected at revision, so please note any specific errors here.

Reviewer #1: (No Response)

Reviewer #2: Yes

6. Review Comments to the Author

Please use the space provided to explain your answers to the questions above. You may also include additional comments for the author, including concerns about dual publication, research ethics, or publication ethics. (Please upload your review as an attachment if it exceeds 20,000 characters)

Reviewer #1: COMMENTS: Although all the comments are answered and few positively attended, frankly speaking I am not very satisfied or convinced about few of the answers/responses [I said ‘FEW of the answers/responses’ about which I am not very satisfied, however, most other responses are alright]. Though, I do not have any specific recommendation, only as system requirement I am choosing ‘major revision’ [assuming that the respected editor would like to give chance to authors for improvement of the manuscript]. Nevertheless, few changes [example: adding (Page 32, Lines 447-452) “Sixth, despite adequate sample size of the two groups based on the initial power calculation, we might also hypothesize that the absence of an effect of the intervention was related to the low number of participants, especially given that smaller effect sizes than expected were observed. The sample size calculation was performed on previous published means related to perceived parental self-efficacy in a population of mothers of term (Leahy-Warren et al., 2012) and preterm (Barnes & Adamson-Macedo, 2007) neonates, which might prove not to have been entirely suitable] are appreciable but let the respected editor decide the future course.

*Response: We appreciate the time you took to look again at our manuscript, and we thank you for acknowledging the changes we made to improve the manuscript’s content.*

*We agree that the limitations need to be carefully acknowledged in this study and they have been listed in the corresponding section pages 31 to 34. Regarding the mention you made about the sample size, we acknowledge that our original power calculation assumed larger effect sizes than we were able to detect in our study. Thus, we added the following statement allowing to better envision future studies (page 32, lines 452-453): “****We therefore recommend that future research is carried out on a larger sample size assuming smaller effect sizes.”***

Reviewer #2: (No Response)

7. PLOS authors have the option to publish the peer review history of their article ([what does this mean?](https://journals.plos.org/plosone/s/editorial-and-peer-review-process#loc-peer-review-history)). If published, this will include your full peer review and any attached files.


If you choose “no”, your identity will remain anonymous but your review may still be made public.


**Do you want your identity to be public for this peer review?** For information about this choice, including consent withdrawal, please see our [Privacy Policy](https://www.plos.org/privacy-policy).

Reviewer #1: No

Reviewer #2: No
